# *In Vivo* Assessment of SMT19969 in a Hamster Model of Clostridium difficile Infection

**DOI:** 10.1128/AAC.02903-14

**Published:** 2014-10

**Authors:** William Weiss, Mark Pulse, Richard Vickers

**Affiliations:** a University of North Texas Health Science Center, Fort Worth, Texas, USA; b Summit plc, Oxfordshire, United Kingdom

## Abstract

SMT19969 [2,2′-bis(4-pyridyl)3*H*,3′-*H* 5,5-bibenzimidazole] is a novel narrow-spectrum nonabsorbable antibiotic currently in development for the treatment of Clostridium difficile infection. The comparative activities of SMT19969 and vancomycin against nonepidemic and epidemic strains of C. difficile were studied in an established hamster model. Against nonepidemic (VA11) strains, the survival rates of SMT19969-treated animals ranged from 80% to 95%. Vancomycin exhibited 100% protection during treatment, with relapse observed starting on day 9 and 50% survival at day 20. At 50 mg/kg of body weight, SMT19969 administered orally once daily for 5 days provided full protection of treated animals on the dosing days and through day 12 against epidemic strains. Vancomycin also protected during the dosing interval, but apparent relapse occurred earlier, starting on day 11. SMT19969 exhibited excellent *in vitro* activity, with MICs of 0.25 μg/ml for all isolates. The MICs for vancomycin were 2- to 4-fold higher at ≤0.5 to 1 μg/ml. All plasma sample concentrations of SMT19969 were below the limit of quantification (25 ng/ml) at all time points, consistent with the reported lack of bioavailability of the compound. Cecal concentrations were significantly above the MIC (ranging from 96 μg/ml to 172 μg/ml).

## INTRODUCTION

In the last decade, Clostridium difficile infection (CDI) has emerged as a significant infectious disease and, with the emergence of hypervirulent strains, an increasing threat in terms of morbidity and mortality (1–3). Although recent increases in disease incidence appear to have leveled off in the United States, CDI was involved in 1% of hospital stays in 2009 (4). In Europe, CDI is associated with estimated costs of 3 billion euros per annum (5). Recurrent CDI adds to the burden of disease, with up to 30% of patients experiencing two or more episodes (6). It is also now recognized that CDI in the community and long-term care facilities may be underreported (6). In addition, when recurrent cases of CDI are included, the incidence in long-term care facilities may exceed that reported in the hospital setting (6). Risk factors for CDI are well documented in the literature and include prior antibiotic use, increasing age, use of proton pump inhibitors, exposure to C. difficile, and underlying gastrointestinal illness (6, 7).

Fidaxomicin, a new compound, was recently licensed for the treatment of CDI but has failed to show superiority over standard therapy for the treatment of hypervirulent strains (8, 9). Therefore, new agents with activity against emerging strains of C. difficile are needed to improve therapeutic options for the treatment of CDI.

**FIG 1:**
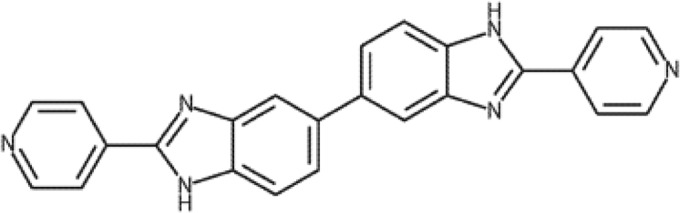
Chemical structure of SMT19969 [2,2′-bis(4-pyridyl)3*H*,3′*H* 5,5-bibenzimidazole].

SMT19969 [2,2′-bis(4-pyridyl)3*H*,3′-*H* 5,5-bibenzimidazole] is a novel nonabsorbable antibiotic (Fig. 1) that inhibits DNA synthesis and is currently in development for the treatment of CDI. With a narrow spectrum of activity and typically >1,000-fold selectivity for C. difficile over Gram-positive and Gram-negative anaerobic and facultative fecal flora (10), SMT19969 has therapeutic potential for the treatment of CDI.

Here, we report the results from studies in an established hamster model (11) of CDI comparing vancomycin with SMT19969.

## MATERIALS AND METHODS

### Antimicrobial agents.

SMT19969 (Summit plc, Oxfordshire, United Kingdom) was formulated in 0.5% methylcellulose (1,500 cP; Sigma). Vancomycin (Hospira, Lake Forest, IL) was reconstituted according to the manufacturer's instructions.

### Bacterial strains.

C. difficile VA11 (nonepidemic restriction endonuclease analysis [REA] J-type strain [UNT103-1]) and VA5 BI/NAP1 (epidemic strain [UNT106-1]) are clinical isolates received from Curtis Donskey (Cleveland VA Hospital, Cleveland, OH) and were previously utilized for the hamster model. Cultures were maintained in a Trypticase soy broth-glycerol stock at −80°C until used.

### MICs.

The MICs for SMT19969, vancomycin, and metronidazole were determined in accordance with National Committee for Clinical Laboratory Standards (2000) methods for antimicrobial susceptibility testing of anaerobic bacteria (12).

### Inoculum preparation.

Freezer stocks of C. difficile were streaked onto Trypticase soy agar (TSA) plus 5% sheep blood (SB) plates 4 days prior to inoculation and anaerobically incubated at 37°C for 48 h. After incubation, the plate growth was suspended into 20 ml of prereduced tryptone-glucose-yeast extract (TGY) (nutrient) broth and anaerobically incubated at 37°C for 24 h. The culture was then diluted 10-fold into SM (sporulation) broth and anaerobically incubated at 37°C for 24 h. On the day of infection (24 h postinoculation of SM broth), the optical density at 600 nm (OD_600_) of the SM broth culture was adjusted to an absorbance of 1.0 (∼1.0E+09 CFU/ml) in prereduced SM broth and then diluted 100-fold in the same medium (∼1.0E+07 CFU/ml).

### Animal model.

All procedures were conducted in accordance with an approved protocol from the University of North Texas Health Science Center IACUC committee. Male golden Syrian hamsters (80 to 120 g) were housed individually with free access to food and water in accordance with NIH guidelines and infected by oral gavage with 0.5 to 0.75 ml of the 100-fold dilution for each organism. The number of CFU input per animal was confirmed by ethanol shock of the OD-adjusted suspension, which was then serial diluted 10-fold in TGY and spot plated onto TSA plus SB (5%) to determine the percentage of spores to vegetative cells. Twenty-four hours after infection (day 0), all animals received a single subcutaneous injection of clindamycin (10 mg/kg of body weight). A further 24 h later (day 1), once-daily administration of SMT19969, vancomycin, or a vehicle was initiated and continued for 5 days.

### Treatment.

**FIG 2:**
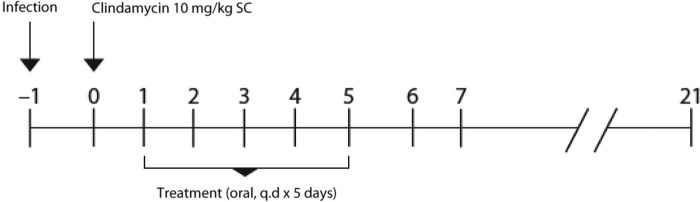
Experimental design and timeline for the hamster model of C. difficile disease. SC, subcutaneously; q.d, once daily.

Hamsters were administered the test compound (Fig. 2) starting 24 h after clindamycin treatment. Single antibiotic doses were administered via oral gavage and formulated in accordance with the sponsors' supplied instructions. Dosing solutions were formulated fresh each day. The antibiotics were administered 1 time per day and continued for up 5 days. The control group was administered the vehicle alone.

### Animal observation.

The animals were observed three times a day up to 21 days postinfection. Any animal judged to be in a moribund state was euthanized.

### Endpoint.

Survival was monitored to day 21.

### Animal disposition.

A necropsy was performed on any animal that was found dead or euthanized during the observation period or at the end of the study; the contents of their cecum were removed, diluted with an equal volume of phosphate-buffered saline (PBS), and frozen at −70°C until processing. Cecum homogenates from animals that died during the study were assayed for the presence of C. difficile toxins A and B using the Wampole C. difficile Tox A/B II enzyme-linked immunosorbent assay (ELISA) kit in accordance with the manufacturer's directions.

### Pharmacokinetics.

Plasma and cecal samples were taken at 1, 3, and 6 h postdose on days 1 and 5 postinfection (three animals per time point) from C. difficile-infected animals and administered 20 mg/kg SMT19969 orally. Cecal samples were extracted two times with an equal volume of methanol, centrifuged to remove cellular debris, and mixed and centrifuged with an equal volume of the internal standard (warfarin), and then 10 μl of the supernatant was injected on a liquid chromatography mass spectrometry (LC-MS) system. Analyses of the plasma and cecal samples were performed at the University of North Texas Health Science Center using adapted LC-MS assay methods (using a Gemini-NX C_18_ 50- by 4.6-mm 3-μl column [Thermo LCQ Deca] for positive electrospray ionization MS).

The data presented are from a pooled analysis of two separate experiments with each C. difficile strain and a total of 15 hamsters assessed per treatment arm overall.

## RESULTS

### C. difficile strain VA11 (UNT103-1).

**FIG 3:**
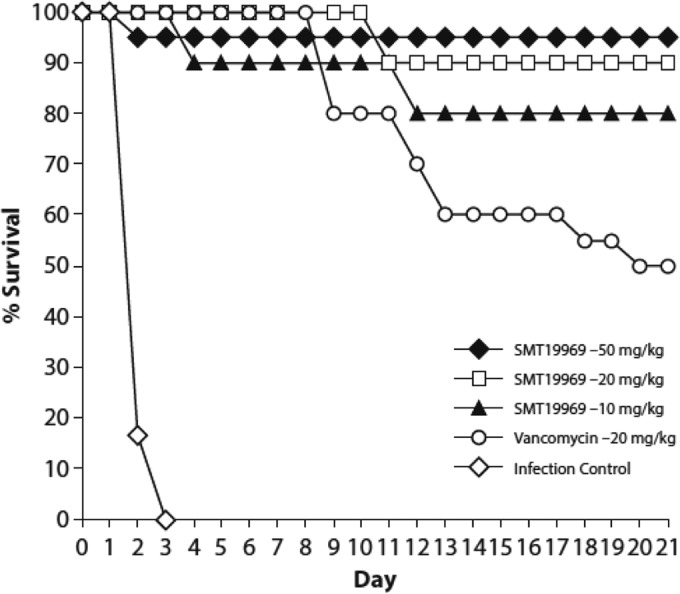
Efficacies of SMT19969 and vancomycin in male golden Syrian hamsters infected with C. difficile strain UNT103-1.

The results of the efficacy study for SMT19969 are presented in Fig. 3. Hamsters were infected with an inoculum of 5.44 to 6.09 log_10_ CFU C. difficile VA11, of which there were 4.59 log_10_ spores contained in the inoculum. Mortality in the untreated control animals occurred between days 1 and 3 of the study, with 0% survival by day 3. For the SMT19969-treated animals, there was one death (on day 2) in the 50-mg/kg dose group (*n* = 20), two deaths (on day 11) in the 20-mg/kg dose group (*n* = 20), and two deaths (on days 4 and 12) in the 10-mg/kg dose group (*n* = 10). No other mortalities were observed in any of the groups, with 95%, 90%, and 80% of the animals surviving at 50, 20, and 10 mg/kg, respectively. Vancomycin provided 100% protection during the treatment, and relapse was observed starting between days 9 and 12 and continuing through day 20 with a 40% to 60% survival rate.

### C. difficile strain VA5 BI/NAP1 (UNT106-1).

**FIG 4:**
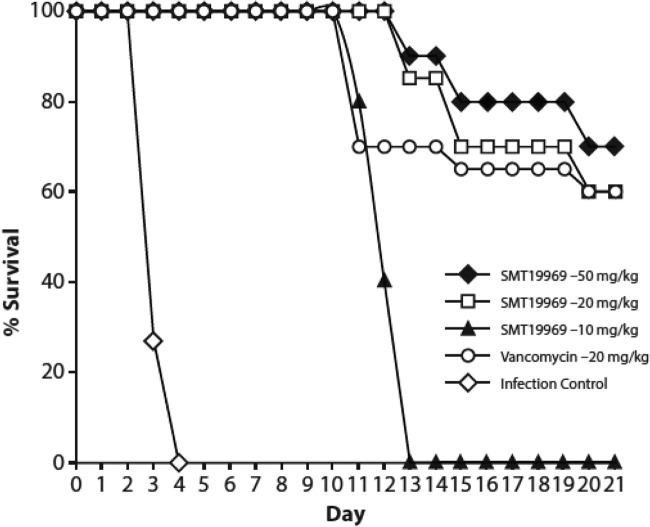
Efficacies of SMT19969 and vancomycin in male golden Syrian hamsters infected with C. difficile strain UNT106-1.

The results of the hamster CDI study of C. difficile VA5 BI/NAP1 administered with SMT19969 and vancomycin are presented in Fig. 4. Hamsters were infected with an inoculum of 5.11 to 6.22 log_10_ CFU of this epidemic C. difficile strain. All untreated controls died between days 2 and 4. SMT19969 at 50 mg/kg, administered orally once daily (q.d.) for 5 days, provided full protection of the treated animals on dosing days and through day 12. One animal died on each of days 13, 15, and 20, with 70% of the animals surviving until the end of the study on day 21. The 20-mg/kg dose of SMT19969 exhibited comparable efficacy and resulted in 60% survival. At 10 mg/kg, SMT19969 provided 100% protection of the infected hamsters during therapy and through day 11 but resulted in 0% survival by day 13. Vancomycin also protected the animals during the dosing interval, but apparent relapse occurred earlier, starting on day 11 and continuing through day 20, with only 60% of the animals surviving to day 21.

### Wampole Tox A/B ELISA results.

With four exceptions, all animals that died on study for both C. difficile strains were positive for C. difficile toxins A/B, whereas all survivors were toxin negative. The exceptions occurred for the VA11 strain with one control animal, two animals treated with 10 mg/kg SMT19969, and one treated with 20 mg/kg SMT19969 that died during the study but exhibited negative toxin results. This was observed in the control and treated groups for VA11-infected animals only, and mortality occurred between days 4 and 12. The reason behind these results is not apparent at this time but may be related to drug effects, gavage error, or sample issues with the toxin assay.

### MIC.

SMT19969 exhibited excellent *in vitro* activity, with an MIC of 0.25 μg/ml for all the isolates, epidemic and nonepidemic. The MICs for vancomycin and metronidazole were at least 2- to 4-fold higher (≤0.5 to 1 μg/ml) than that for SMT19969.

### Pharmacokinetics.

Results of analysis of the plasma and cecal samples from infected hamsters administered 20 mg/kg SMT19969 orally once daily for 5 days are presented in Table 1. SMT19969 concentrations in all of the plasma samples were below the limit of quantification (25 ng/ml) for the three time points on days 1 and 5 of dosing, consistent with the reported lack of bioavailability of the compound. The observed mean cecal concentrations were 95.7, 172.2, and 145.8 μg/ml at 1, 3, and 6 h posttreatment on day 1. Levels of 109.3, 146.4, and 130.3 μg/ml were determined for the same time points on day 5 following the 5 days of dosing.

**TABLE 1:** Pharmacokinetics of SMT19969 in plasma^*a*^ and cecal contents of male hamsters following oral administration once per day for 5 days

Day	Time (h)	SMT19969 concn (μg/ml) in ceca	
		Mean	SD
1	1	95.7	40.6
	3	172.2	23.7
	6	145.8	90.6
5	1	109.3	53.5
	3	146.4	87.9
	6	130.3	142.7

a SMT19969 concentrations were below the limit of quantification (25 ng/ml) in all plasma samples.

## DISCUSSION

The hamster model of clindamycin-induced CDI is the current standard *in vivo* model used to assess the potential efficacies of agents, including antibiotics, toxin antibodies, and vaccines (10, 11). The model has also been used to study the virulence and disease pathogenesis of CDI (13, 14). The model replicates many of the features of human disease, with C. difficile toxin-mediated inflammation of the gastrointestinal (GI) tract resulting in histopathological changes in the tissue comparable to those observed in humans (11). This model has shown that toxins A and B, as well as the newly identified C. difficile toxin (CDT) or binary toxin, may be equally important in terms of the etiology of CDI and the severity of infection, although further work is still needed to fully understand the role of toxins in the disease process (16). Recent efforts to more closely replicate human disease have resulted in models in other species, including a murine model that shows less acute symptoms that more closely mimic those in humans (16). However, the hamster model remains the standard model in which to assess the basic efficacies of novel CDI agents, and results from studies using this model are generally considered predictive of their efficacy in humans.

SMT19969 is a novel antibiotic in clinical development for the specific treatment of CDI that has potent growth inhibition of C. difficile (MIC_90_, 0.125 μg/ml) and is associated with minimal growth inhibition of organisms comprising the normal healthy human gut microbiota (10). MIC susceptibility testing of SMT19969 against the C. difficile strains used in these studies showed good growth inhibition, with an MIC of 0.25 μg/ml, which was 2- to 4-fold more potent than those recorded for vancomycin or metronidazole. SMT19969 has also been shown to be 1 dilution more active against C. difficile isolates (MIC range, 0.125 to 0.5 μg/ml; MIC_90_, 0.25 μg/ml), including ribotype 027, than fidaxomicin (MIC range, 0.06 to 1 μg/ml; MIC_90_, 0.5 μg/ml) (10). In addition, SMT19969 has a favorable pharmacokinetic profile, with high levels being achieved in the GI tract following oral administration and minimal systemic exposure (10).

C. difficile VA11 (UNT103-1) is an REA J-type clinical isolate responsible for outbreaks of C. difficile infection in North America (17). Use of this strain in the hamster model showed a reproducible recurrence event in vancomycin-treated animals, with mortality typically initiating around day 12 postinfection. Results with SMT19969 administration were encouraging; hamsters were protected from mortality during the course of dosing and from recurrent disease during the follow-up period (Fig. 3). At doses of either 20 mg/kg or 50 mg/kg, SMT19969 showed efficacy superior to that of vancomycin, with 80% to 95% survival rates recorded on day 21 over the three dose levels of SMT19969 compared to only a 50% pooled survival rate for animals treated with vancomycin (20 mg/kg). The acute nature of C. difficile infection in hamsters was demonstrated in the vehicle control groups, with a pooled mortality rate of 100% by day 3.

SMT19969 also exhibited efficacy in the hamster model against the epidemic VA5 BI/NAP1 strain (UNT106-1). The low 10 mg/kg dose provided protection from the infection during dose administration, with relapse starting at day 11. SMT19969, administered at 50 mg/kg, exhibited the greatest efficacy of all the dose groups, with higher overall survival and greater time to observed mortality. The 20-mg/kg dose of SMT19969 and of vancomycin provided comparable protection at the end of the study (60% survival), but the time to relapse was delayed for the animals given SMT19969 compared to those administered vancomycin.

The concentrations of SMT19969 (20 mg/kg) in the plasma and cecal contents of C. difficile-infected animals on days 1 and 5 of dosing were assessed. Concentrations were below the limit of quantification (25 ng/ml) in all of the plasma samples, indicating minimal systemic exposure of SMT19969 following oral dosing. At 1, 3, and 6 h postdose, cecal concentrations were significantly higher than the MIC (ranging from 96 to 172 μg/ml), and no significant accumulation of the drug was observed.

SMT19969 has demonstrated a narrow and highly selective spectrum of activity against C. difficile and has been shown to be contained within the gut, thus enabling high concentrations of SMT19969 in relation to the MICs for C. difficile and leaving undetectable levels in plasma and GI tissue. The efficacy of the study compound compared with that of the current standard of care, vancomycin, has been substantiated and shown to be superior in the hamster model, as evidenced by greater overall survival, delayed time to relapse, and lower recurrence of infection. Clinical trials in humans with SMT19969 are now warranted.
